# Complement-Amplifying Conditions in Atypical Hemolytic Uremic Syndrome: A Canadian Case Series

**DOI:** 10.1177/20543581221100288

**Published:** 2022-05-19

**Authors:** Christopher J. Patriquin, Katerina Pavenski, Jocelyn Garland, Louis-Philippe Girard, Paul Isenring

**Affiliations:** 1 Division of Medical Oncology & Hematology, University of Toronto, ON, Canada; 2 St. Michael’s Hospital, Unity Health Toronto, University of Toronto, ON, Canada; 3 Division of Nephrology, Department of Medicine, Queen’s University, Kingston, ON, Canada; 4 Division of Nephrology, Foothills Medical Centre, University of Calgary, AB, Canada; 5 Nephrology Research Group, Department of Medicine, L’Hôtel-Dieu de Québec Institution, Laval University, Québec, QC, Canada

**Keywords:** complement pathway, hemolytic uremic syndrome, organ dysfunction, thrombotic microangiopathy, thrombotic thrombocytopenic purpura

## Abstract

**Rationale:**

Thrombotic microangiopathies (TMAs) are systemic disorders that often affect the kidneys and encompass a heterogeneous group of conditions, including atypical hemolytic uremic syndrome (aHUS). The complement pathway is thought to play a crucial role in the pathogenesis of aHUS, and a favorable response can be obtained through complement C5 inhibition. There is emerging evidence to suggest that the same is also true for several other forms of TMA.

**Objective:**

The purpose of this series is to report cases of aHUS in which both an innate defect of the alternative complement pathway and a complement-amplifying condition were suspected.

**Methods:**

This case series describes 8 patients who were managed in Canadian tertiary centers for aHUS and who presented initially with complement-amplifying conditions.

**Results:**

In all cases, aHUS was associated with organ dysfunction and in some, with an innate defect of the alternative complement pathway. The complement-amplifying conditions identified were diverse including immune disorders, pregnancy, and a *Shiga* toxin infection. Patients improved rapidly when treated with eculizumab or plasma exchange.

**Conclusions:**

These observations illustrate the seriousness of secondary aHUS. They also add to existing lines of evidence that the complement pathway is potentially involved in this condition and that it should be considered as a therapeutic target of interest under such circumstances.

## Abrégé

**Justification:**

Les microangiopathies thrombotiques (MAT) sont des troubles systémiques qui affectent souvent les reins et qui englobent un groupe hétérogène d’affections, notamment le syndrome hémolytique et urémique atypique (SHUa). On pense que la voie du complément joue un rôle crucial dans la pathogenèse du SHUa et qu’une réponse favorable pourrait être obtenue par inhibition du complément C5. De nouvelles preuves suggèrent qu’il en serait de même pour plusieurs autres formes de MAT.

**Objectif:**

Cette série vise à rapporter des cas de SHUa pour lesquels on soupçonnait à la fois une anomalie congénitale de la voie alterne du complément et une condition d’amplification du complément.

**Méthodologie:**

Cette série décrit les cas de huit patients qui présentaient initialement des conditions d’amplification du complément et qui ont été pris en charge pour un SHUa dans des centres tertiaires canadiens.

**Résultats:**

Dans tous les cas, le SHUa était associé à un dysfonctionnement d’organe et, dans certains cas, à une anomalie congénitale de la voie alterne du complément. Les conditions d’amplification du complément identifiées étaient diverses, notamment des troubles immunitaires, une grossesse et une infection à une shigatoxine. L’état des patients s’est rapidement amélioré après un traitement avec éculizumab ou des échanges plasmatiques.

**Conclusion:**

Ces observations illustrent la gravité du SHUa secondaire. Elles s’ajoutent aux preuves existantes qui suggèrent que la voie du complément est potentiellement impliquée dans cette pathologie et qu’elle devrait être considérée comme une cible thérapeutique d’intérêt dans de telles circonstances.

## Introduction

Thrombotic microangiopathy (TMA) refers to a histopathologic lesion of the arteriole or capillary that consists of platelet-rich fibrinous thrombi with tumefaction of the endothelium.^1^ It causes consumptive thrombocytopenia with mechanical hemolytic anemia and ischemic damage in affected tissues.

TMAs are now often categorized into 3 groups as follows: (1) thrombotic thrombocytopenic purpura (TTP) due to a deficiency in a disintegrin and metalloproteinase with a thrombospondin type 1 motif, member 13 (ADAMTS13) activity, (2) typical hemolytic uremic syndrome (HUS) due to *Shiga* toxin (STX)-producing *Escherichia coli* (STEC) infections, and (3) atypical HUS (aHUS), a disorder that is categorized further into primary and secondary aHUS (associated with miscellaneous conditions as listed in Table 1).^1-3^

**Table 1.** Conditions Associated With Secondary Thrombotic Microangiopathy.^1-3^

Groups	Disorders
Autoimmune	Systemic sclerosis/scleroderma Anti-phospholipid antibody syndrome Systemic lupus erythematosus Primary glomerulonephritis
Cancers	Solid organ cancers Bone marrow transplant
Drugs	Calcineurin inhibitors Quinine Interferon Tyrosine kinase inhibitors Oral contraceptives Mitomycin, gemcitabine
Pregnancy and post-partum	Hemolysis, elevated liver enzymes and low platelet count Pre-eclampsia/eclampsia
Infections	Cytomegalovirus Hepatitis C virus Human immunodeficiency virus H1N1 influenza A subtype BK virus
Severe hypertension	
Organ transplantation	

*Note.* This list shown is not exhaustive.

The group *KDIGO* (“Kidney Disease: Improving Global Outcomes”) recently coined the term “primary aHUS” to encompass those forms of TMA resulting from complement gene defects (see list in Table 2).^4^ However, there is evidence to suggest that such defects can also occur in STEC-HUS, secondary aHUS, or TTP.^7^ Some patients with STEC-HUS and secondary aHUS may also have complement-mediated damage to the endothelium.^1,2,7^

**Table 2.** Genes Involved in aHUS.

Causative^4^	Candidate^5,6^
CFH	*C9*
*CFHR1* ^a^	*PLG*
*CFHR2* ^a^	*F12*
*CFHR3* ^a^	*FKRP*
*CFHR4* ^a^	*INF2*
*C3*	*ST3GAL1*
*CFI*	*VWF*
*CD46*	*CR1*
*THBD* *DGKE* *MMACHC*	*VTN* *CFB* *CFD*

*Note.* aHUS = atypical hemolytic uremic syndrome; *C9* = complement component 9; *CFHR* = complement factor H-related protein; *PLG* = plasminogen; *F12* = coagulation factor XII gene; *FKRP* = fukutin-related protein gene; *INF2* = inverted formin-2; *C3* = complement component 3; *ST3GAL1* = ST3 Beta-Galactoside Alpha-2,3-Sialyltransferase 1; CFI = complement factor I; *VWF* = von Willebrand factor; *CD46* = cluster of differentiation 46; *CR1* = complement C3b/C4b receptor 1; *THBD* = gene encoding for thrombomodulin; *VTN* = vitronectin; *DGKE* = diacylglycerol kinase epsilon; CFB = complement factor B; *MMACHC* = methylmalonic aciduria and homocystinuria type C.

a The genes are considered risk factors for complement-mediated disorders through deletion of various segments within the *CFHR1* to *CFHR4* locus.

In both primary and secondary aHUS, a triggering event often precedes microvascular injury and can itself act as a condition that amplifies the complement pathway afterwards.^8,9^ As HUS appears to be genetically determined in many cases, TMA could thus result from an abnormal host-response to an environmental factor. As for many other conditions, however, the relative importance of predisposing versus acquired factors in TMA-causing disorders has only been assessed in a limited number of studies.^10^

Here, we report cases of aHUS in which a complement-amplifying condition (CAC) was suspected, pathogenic or risk variants in associated genes were identified (4 out of 8 patients), and anti-complement-based interventions led to clinical improvement. The cases described provide further evidence that a vicious cycle of complement activation could be a common pathogenic trait in many forms of HUS.

## Methods

Eight patients who presented with signs of TMA in the setting of a CAC and who were subsequently diagnosed with aHUS are described. A summary of the routine laboratory and genetic data obtained in each case is shown in Tables 3 and 4, respectively. When patients were started on eculizumab, they were also treated with prophylactic anti-infectious regimens.^11^ All patients included in this case series gave written informed consent for publication of their clinical and biological data.

**Table 3.** Blood Results of the Patients Included in This Series.

	Hb (g/L)	PLTs (10^9^/L)	SCr (μM)	Haptoglobin	LDH (u/L)	ADAMTS13	Schistocytes (+/−)
Case 1	79	65	200	U	1194	N	+
Case 2	82	20	140	U	1113	N	+
Case 3	93	36	592	U	2873	N	+
Case 4	68	118	774	U	480	N	+
Case 5	79	117	675	U	408	N	−
Case 6	79	124	187	U	380	N	−
Case 7	103	22	264	N/A	1153	N/A	+
Case 8	69	52	503	N	266	N	N/A

*Note.* All results were obtained at the time of presentation or after referral. Note that the values of LDH shown were all above normal. Hb = hemoglobin; PLTs = platelets; SCr = serum creatinine; LDH = lactate dehydrogenase; ADAMTS = a disintegrin and metalloproteinase with a thrombospondin type 1 motif; U = undetected; N = normal; N/A = not available.

**Table 4.** Genes Sequenced for the Patients Included in This Series.

	Cases							
	1^a^	2^b^	3^a^	4^b^	5^b^	6^b^	7^b^	8^b^
Genes tested								
*CFH*	•	•	•	•	•	•	•	•
*CFHR1*	•		•					
*CFRH2*	•		•					
*CFHR3*	•		•					
*CFHR4*	•		•					
*CFHR5*	•	•		•	•	•	•	•
*CFI*	•	•	•	•	•	•	•	•
*C3*	•	•	•	•	•	•	•	•
*CD46*	•	•	•	•	•	•	•	•
*THBD*	•	•	•	•	•	•	•	•
*DGKE*			•					
*CFB*	•	•	•	•	•	•	•	•
*CFD*			•					

*Note. CFHR* = complement factor H-related protein; CFI = complement factor I; *C3* = complement component 3; *CD46* = cluster of differentiation 46; *THBD* = gene encoding for thrombomodulin; *DGKE* = diacylglycerol kinase epsilon; CFB = complement factor B.

a DNA testing was carried out by next generation sequencing and analyzed for copy number variation.

b DNA testing was carried out through conventional Sanger sequencing.

## Results

### Case 1

A 79-year-old male with known stage-II chronic kidney disease presumed to be age-related presented with worsening fever, chills, shortness of breath, and nonproductive cough despite treatment with amoxicillin. He was found to have eosinophilic pneumonia and started on steroids as well as broad-spectrum antibiotics. On admission, his serum creatinine (SCr) was 65 μmol/L and estimated glomerular filtration rate (eGFR) was 77 mL/min/1.73 m^2^.

A few days following admission, a magnetic resonance imaging (MRI) scan ordered for obtundation showed multiple small-sized cerebral emboli. Additional blood tests also showed the following results: thrombocytopenia and microangiopathic hemolytic anemia (MAHA; Table 3), SCr 200 μmol/L (eGFR 25 mL/min/1.73 m^2^), complement component 3 (C3), C4 and ADAMTS13 activity normal, absence of monoclonal (M) spike by serum and urine electrophoresis, anti-nuclear antibodies or extractable nuclear antigens below threshold, and human immunodeficiency virus negative.

A renal biopsy carried out in this context showed evidence of acute and widespread glomerular and periglomerular TMA superimposed on a chronic but milder component of chronic TMA in the form of ischemic tuft shrinkage and segmental basement membrane duplication. There were no signs of glomerulonephritis based on immunofluorescence and electron microscopy.

The patient was thus diagnosed with subacute aHUS in the setting of eosinophilic pneumonia. In addition to the antibiotics, he received 5 sessions of plasma exchange (PLEX) and, in the absence of improvement, was treated with eculizumab 3 weeks after admission. He experienced full hematologic remission 2 weeks later and his eGFR increased to 50 mL/min/1.73 m^2^. The patient was discharged on maintenance eculizumab and remains in remission at the time of writing.

Further investigation led to the identification of a *CFHR3-1* heterozygous deletion (Table 4). This abnormality is generally not considered pathogenic by itself and is relatively common in the general population. However, it is more commonly seen in aHUS and could thus have played an important role in the development of TMA.^8,12^ In addition to the severity of the clinical presentation and presence of chronic TMA on the renal biopsy, the genetic variant identified was an incentive to pursue eculizumab over the long term.

### Case 2

A 68-year-old female presented with severe diarrhea, and decreased level of consciousness of recent onset. She had been experiencing unexplained cognitive impairment during the last year and said to have had developed dementia. Except for receiving treatment for hypertension and hypothyroidism, her past medical history was unremarkable.

An initial blood workup showed acute kidney failure with a SCr of 140 μmol/L (eGFR 32 mL/min/1.73 m^2^), severe proteinuria, thrombocytopenia and MAHA (Table 3). Additional tests revealed the following results: ADAMTS13 activity normal, C3 and C4 serum levels also normal, M spike undetected by serum or protein electrophoresis, and autoimmune serology unexpectedly active with elevated rheumatoid factor, anti-SSA/Ro antibodies, anti-nuclear antibodies, and anti-cyclic citrullinated peptide. No abnormalities were seen on a brain MRI and no signs of malignancy on imaging studies either. A kidney biopsy showed findings of acute TMA with diffuse C5b-9 deposition in glomerular capillaries, but no evidence of glomerulonephritis.

The patient was diagnosed with aHUS in the setting of a possible, but undefined connective tissue disease. PLEX therapy was initiated without improvement. Management was thus escalated to eculizumab and the hematologic parameters improved rapidly. Several days after admission, pre-PLEX blood tests revealed normal complement factor H (CFH), negative anti-CFH antibody titers, and low complement factor B (CFB) with elevated Bb fragment and C5b-9. An in vitro C5b-9 deposition test carried out on endothelial cells by Noris et al^13^ was markedly abnormal initially, but normalized a few weeks later under eculizumab therapy. Although the cognitive status of the patient improved, eculizumab was stopped as care was transitioned to palliative because of permanent need for dialysis.

The patient was eventually subjected to genetic testing of the alternative complement pathway (Table 4). However, no genetic variants of importance could be identified. Of notice, however, the CFHR1, CFHR2, and CFHR3 genes were not included in the analysis.

### Case 3

A 68-year-old male was admitted for HUS complicated by acute oliguric renal failure with a SCr of 592 μmol/L (eGFR <15 mL/min/1.73 m^2^). Four days previously, he had begun experiencing nonbloody diarrhea after returning from Cuba. Five months before, SCr level was 99 μmol/L (eGFR 61 mL/min/1.73 m^2^). Medical and therapeutic histories were otherwise unremarkable.

On arrival, blood pressure was severely increased, jugular vein distended, and ankles swollen. A blood workup showed thrombocytopenia and MAHA (Table 3). In addition, fibrinogen was increased, and international normalized ratio (INR) and free κ/λ ratio were normal. Hemodialysis, PLEX therapy, and steroids were started soon after admission, but were discontinued later on upon return of a normal ADAMTS13 activity assay.

Several days after admission, blood results for CFH, CFB, and plasminogen activity returned normal, but a stool specimen tested positive for STX2 *Shiga* toxin. The patient was thus diagnosed with STEC-HUS and treated supportively. He was not considered for eculizumab in this context and given that other infections had complicated the hospital course. After 5 weeks, the patient made a full recovery.

Two months after admission, genetic tests showed that the patient carried a heterozygous Ile221Val substitution in complement *CFH* (Table 4). This variant is considered to be a risk allele for the development of complement-related disorders.^14^

### Case 4

A 37-year-old male was admitted with bilateral leg edema and intermittent brown urine. Medical history included ulcerative colitis, gastroesophageal reflux, and chronic hepatitis B that had been diagnosed 2 years before. Family history was unremarkable.

Blood tests revealed a SCr of 202 μmol/L (eGFR 30 mL/min/1.73 m^2^) as well as slightly decreased hemoglobin (Hb), albumin, and C3 levels (Table 3). Autoimmune serology was negative. A renal biopsy revealed mild proliferative crescentic glomerulonephritis, mesangial and capillary C3 deposition on immunofluorescence, and dense deposits on electron microscopy. There was no evidence of TMA. The patient was diagnosed with C3-dominant postinfectious glomerulonephritis and started on tenofovir for hepatitis B. However, it was not clear that the infection was directly at cause given that it had antedated the renal disease by several months. After 6 weeks, the antiviral agent was discontinued due to intolerance.

Six months after the first admission, the patient presented with persistent nausea, progressive fluid overload, mild hypertension, and reduced urine output due to severe renal failure. SCr had progressively increased to 774 μmol/L (eGFR <15 mL/min/1.73 m^2^). The patient required hemodialysis temporarily. Further investigation revealed thrombocytopenic MAHA with normal ADAMTS13 activity and STX-negative stools. There was no M spike by serum electrophoresis and autoimmune serology was negative. A repeat renal biopsy revealed TMA on a background of chronic C3 glomerulopathy, an association that has been described on repeated occasions in the past.^4,15^

The patient was diagnosed with aHUS superimposed on another complement-related disorder. He was started on PLEX therapy initially and on eculizumab therapy 2 months after admission while receiving lamivudine in prophylaxis of a hepatitis B flare. Hematologic parameters normalized progressively in the following months and SCr stabilized to a new baseline of approximately 150 μmol/L (eGFR ~50 mL/min/1.73 m^2^). No genetic variants of importance or anti-CFH antibodies were detected a posteriori (Table 4). For this reason, and because HUS was no longer active clinically, eculizumab was discontinued 18 months after initiation.

### Case 5

After a curettage procedure for a first trimester miscarriage, a 25-year-old female developed postoperative endometritis with acute kidney injury (SCr 675 μmol/L; eGFR <15 mL/min/1.73 m^2^), hypertension, mild thrombocytopenia, and MAHA (Table 3). Liver enzymes, INR, and serum electrophoresis were normal. Blood cultures were negative and there was no evidence of infection otherwise. The patient was diagnosed with aHUS and subsequently found to carry a *C3* variant of unknown significance on genetic tests.

The patient was started on hemodialysis and PLEX therapy soon after the curettage procedure. Platelet count and lactate dehydrogenase (LDH) levels rapidly returned to normal. Three weeks later, eculizumab was initiated and plasma therapy maintained for another 3 weeks until renal function normalized.

The patient remained on eculizumab for 1 year. She was seen a year later when she suffered another first-trimester miscarriage, but with no clinical evidence of recurring aHUS. Hematologic parameters remained stable during this episode. Eculizumab was not reinstated as aHUS was no longer active and as genetic testing had revealed inconclusive (Table 4). However, complement C5 inhibition will be considered should the patient plan for another pregnancy.

### Case 6

An 18-year-old male was admitted with deteriorating renal function. He had a medical history of chronic antiphospholipid syndrome diagnosed at age 14 after an episode of spontaneous deep vein thrombosis with pulmonary embolism, and he was receiving warfarin chronically because of this complication. He was also treated with steroids for systemic lupus erythematosus that was diagnosed in the setting of recurrent seizures.

On admission, laboratory tests revealed a SCr of 187 μmol/L; eGFR 42 mL/min/1.73 m^2^, mild thrombocytopenia in the absence of schistocytes, negative direct antiglobulin test (DAT), and normal ADAMTS13 activity (Table 3). Genetic testing (Table 4) showed that the patient carried a heterozygous pathogenic variant in *CFB* (c.1598A>G) and a variant of unknown significance in *C3* (c.4716C>T). The patient was diagnosed with aHUS on a background of autoimmune disease.

Eculizumab was thus initiated while warfarin and prednisone were continued. PLEX was not administered due to immediate availability of eculizumab. The patient was eventually switched from warfarin to rivaroxaban following a cerebral thrombotic event. After 5 years, he is still on eculizumab with stable renal function (sCR 140 µM; eGFR 60 mL/min) and hematologic parameters (platelets generally >100 × 10^9^/L). He most likely will be maintained on C5 inhibition indefinitely given the severity of the clinical presentation and the genetic variant identified.

### Case 7

A 41-year-old female was referred to the clinic for follow-up of previously diagnosed TTP. Her past medical history was otherwise unremarkable except for hypothyroidism and a pregnancy at age 34.

Between ages 27 and 35, she experienced 5 episodes of thrombocytopenic MAHA with severe acute renal failure. The first episode occurred 2 weeks after vaccination for influenza, the second during a febrile viral illness, the third near a pregnancy term, the fourth in the absence of any apparent trigger, and the fifth following pneumonia.

During all episodes, the patient received high-dose steroids and PLEX therapy. She recovered fully each time except during the fourth episode, for which she also received rituximab and underwent splenectomy, and during which she had normal ADAMTS13 activity as tested on 2 occasions. After the fifth episode, she was left with chronic renal failure, anemia, and high blood pressure.

Her other laboratory values from the last episode are provided in Table 3. While in remission afterwards, C3 and CH50 serum levels were found to be low, but C4 serum levels were normal and lupus anticoagulant as well as anti-phospholipid antibodies were negative. Genetic analyses (Table 4) eventually revealed a pathogenic heterozygous variant in *CFI* (c.949C>T; p. Arg317Trp). Taking into account this genetic variant, a clinical presentation that was consistent with relapsing complement-mediated aHUS and substantial baseline renal dysfunction, treatment with eculizumab was begun.

Fourteen years after presentation, the diagnosis of TTP was written off as being wrong. The patient has experienced no recurrence of thrombocytopenic MAHA since starting on eculizumab and is currently doing well. There is no plan to discontinue C5 inhibition for the time being.

### Case 8

A 41-year-old female kidney transplant recipient on cyclosporine was transferred to our hospital for biopsy-proven renal TMA of her allograft and for diffuse alveolar hemorrhage requiring mechanical ventilation. The graft had been donated by her bother 8 years earlier and had remained functionally stable afterwards. The patient was also known for severe Crohn’s disease since age 15 and had been experiencing exacerbation several weeks prior.

At age 26, 6 months after an uncomplicated pregnancy, the patient developed a viral illness with nonbloody diarrhea and hematuria. Two weeks later, she was found to have thrombocytopenic MAHA with severe renal failure (SCr 588 µM; eGFR < 15 mL/min) and biopsy-proven TMA in her native kidney. The patient was treated with high-dose steroids, plasmapheresis, and temporary hemodialysis. Over the following months, she developed worsening renal failure and congestive heart failure with severe mitral regurgitation. She eventually underwent mitral valve repair and required hemodialysis for 8 years until she received the kidney graft.

Upon hospitalization for the episode of alveolar hemorrhage, Hb was 69 g/L, platelets were 52 × 10^9^/L, serum LDH was mildly elevated, SCr was 503 μmol/L (eGFR < 15 mL/min), and serum C3 levels were low. Autoimmune serology was negative. The patient was started on hemodialysis, high-dose steroids, and PLEX. Both the respiratory and hematologic condition improved initially, but deteriorated once again upon treatment cessation. After receiving a single dose of eculizumab, the patient went into remission and was discharged on chronic hemodialysis. This drug was not pursued beyond the first dose in the absence of private or public coverage but will be reinstated long term in case of recurrence or if the patient receives another kidney transplant.^16,17^

Genetic studies (Table 4) later revealed that the patient was heterozygous for an unreported variant in the *CFHR5* gene (c.1412G>A; p. Gly471Glu). This variant is predicted to be pathogenic based on in silico analyses. However, it was considered of uncertain clinical relevance as thrombocytopenic MAHA had not recurred early on after the renal transplant. The overall clinical and biological presentation was still consistent with complement-mediated aHUS. Cyclosporine was also unlikely to be the cause of the latest deterioration as the patient had already been on this medication for 8 years.

## Discussion

Our patients were all affected by severe forms of aHUS that led to permanent organ dysfunction in many. All, if not most, were affected by an underlying condition that could have acted as a coexisting CAC, that is, by eosinophilic pneumonia, an autoimmune disorder, a complement-mediated glomerular disease, pregnancy, and a STX-related diarrheal illness. The cases described were thus consistent with secondary aHUS.

Complement dysfunction was also suspected in all of our patients. Aside from the associated CAC, it was indeed suggested by at least 1 (typically 2) of the following features in each case: (1) low C3 serum levels, (2) C5b-9 deposition, (3) a pathogenic variant in a complement gene, and (4) a clear response to eculizumab or PLEX. These observations illustrate the limitations of the current TMA classification from both the pathophysiologic and therapeutic perspectives. In secondary aHUS, for instance, consumptive thrombocytopenia and organ dysfunction can improve not only by treating the triggering condition but also by inhibiting the complement pathway.^3^

There is in fact clear evidence to suggest that the alternative and terminal complement pathways can play a pathogenic role in many forms of aHUS.^3,9,18,19^ The same appears to hold true for TTP. For instance, primary aHUS has been linked to von Willebrand factor (*VWF*),^20,21^ a coagulation factor that is involved in the development of TTP and that can interact with CFH.^22^ Along the same line, the multicomponent protein network of the coagulation pathway is interlinked with the complement through several crossover points.^23^ It is perhaps for this reason that gene defects in thrombomodulin can lead to aHUS.^24,25^

When the complement pathway is activated in aHUS, pathogenic variants in 1 or several genes that sustain or regulate C5b-9 activity can lead to uncontrolled deposition of the attack complex and subsequent endothelial damage. The frequency of such variants in the pediatric and adult population has been reported in certain series to be as high as 60-70%^26,27^ and even higher in some forms of secondary aHUS.^28^ In the adult population, however, it was reported in one series to be much lower in primary and secondary aHUS,^29^ and in 2 others, to be much lower in secondary aHUS.^19,30^

The importance of triggering events in the development of TMA (events that are referred to as “hits” by Riedl and colleagues)^9^ has led to the concept of CAC, whereby the natural propensity of the complement to undergo auto-amplification is enhanced. Because auto-amplification promotes the formation of C3bBb-properdin complexes through which C3b is released from both the spontaneous form of C3 (C3·H_2_O) and induced form of C3, a CAC could potentially act by cleaving C3·H_2_O directly or increasing properdin activity.^31^

Examples of CACs include viral and bacterial infections, pregnancy, systemic lupus erythematosus, bone marrow transplant, and drugs.^2,8,10,32^ In some previously reported cases, STEC infection was even implicated as a CAC while this condition has been said to elicit TMA in the absence of predisposing abnormalities.^3,7,9^ One must also remember that primary aHUS not uncommonly presents initially as a condition that can be unmasked by a CAC.^1^

CACs may also correspond to the main cause of TMA rather than the triggering event per se. Making the distinction among these designations, that is, CAC, cause, and trigger, is not always easy, but is still an important step to take for the diagnosis and management of these disorders. It should also allow one to consider the relative importance of acquired versus predisposing factors and guide therapy.

On the practical side, a decrease in ADAMTS13 activity and the presence of STX1 and STX2 in stools should be looked for early on during the course of a TMA episode to rule out TTP and STEC-HUS. In their absence, several disorders or factors (Table 1) could be at cause, and those likely to be involved in the development of TMA should be acted upon specifically. It is still unclear based on the studies available^19,30^ that eculizumab can be of benefit under such circumstances. In our opinion, however, it could serve as a useful adjunct, at least temporarily, if complement involvement is suspected and if the underlying cause cannot be treated rapidly or severe organ dysfunction is present.^2,33^

As demonstrated through the cases presented in this series, investigating the complement pathway in most forms of TMA should be also seen as probably essential (Table 5). However, it is well established that the traditional markers of complement activation, such as C3, CH50 and AH50, are not sensitive in aHUS. For this reason, many clinicians now use more refined serum markers (see Table 5) and in vitro (C5b-9 deposition) assays even if the usefulness of these other investigative approaches has not yet been validated.^29^ To ensure that a triggering event or CAC is not mistaken for an actual cause, many clinicians also investigate many forms of TMA through genetic tests (Tables 2, 4, and 5).

**Table 5.** Recommended Investigation in Cases of ADAMTS13-Negative MAHA or Biopsy-Proven TMA.

Blood
C3, C4, Ba, or Bb
CH50, AH50
CFH, CFI
Anti-CFH and anti-CFI
C3NEF
sC5b-9
Genetic tests
Tissues
C5b-9 deposition^a^
C3b deposition assay^b^

Whenever possible, a functional rather than quantitative assay should be obtained. MAHA = microangiopathic hemolytic anemia; TMA = thrombotic microangiopathies; C3 = complement component 3; C4 = complement component 4; CH50 = 50% hemolytic complement; CFH = complement factor H; CFI = complement factor I; C3NEF = C3 nephritic factor; sC5b-9 = serum complement membrane attack complex; HUS = hemolytic uremic syndrome.

a Assessed by immunohistochemistry or immunofluorescence in a tissue affected by TMA.

b Assayed by certain research laboratories in commercial endothelial cell layers incubated with the serum of an affected patient.^13,34^ Note that many of these tests are not highly sensitive for HUS or still await validation as to their diagnostic usefulness.

## Conclusions

The cases presented in this report all appeared to have been affected by secondary aHUS as defined in the literature, but also required PLEX or eculizumab for their clinical condition to improve. Perhaps, more specific and well-defined disease terminology could be beneficial according to these observations. In particular, the decision to treat most forms of TMA through agents such as eculizumab should probably be based on evidence of complement dysregulation and the importance of organ dysfunction.
